# Data on impact of monocytes and glucose fluctuation on plaque vulnerability in patients with coronary artery disease

**DOI:** 10.1016/j.dib.2018.03.022

**Published:** 2018-03-10

**Authors:** Hiroyuki Yamamoto, Naofumi Yoshida, Toshiro Shinke, Hiromasa Otake, Masaru Kuroda, Kazuhiko Sakaguchi, Yushi Hirota, Takayoshi Toba, Hachidai Takahashi, Daisuke Terashita, Kenzo Uzu, Natsuko Tahara, Yuto Shinkura, Kouji Kuroda, Yoshinori Nagasawa, Yuichiro Nagano, Yoshiro Tsukiyama, Ken-ichi Yanaka, Takuo Emoto, Naoto Sasaki, Tomoya Yamashita, Wataru Ogawa, Ken-ichi Hirata

**Affiliations:** a Division of Cardiovascular Medicine, Department of Internal Medicine, Kobe University Graduate School of Medicine, 7-5-1 Kusunoki-cho, Chuo-ku, Kobe 6500017, Japan; b Division of Diabetes and Endocrinology, Department of Internal Medicine, Kobe University Graduate School of Medicine, Kobe, Japan

## Abstract

Data presented in this article are supplementary material to our research article entitled “Impact of CD14^++^CD16^+^ monocytes on coronary plaque vulnerability assessed by optical coherence tomography in coronary artery disease patients” [1]. This article contains the data of study population, diagnostic ability of CD14^++^CD16^+^ monocytes to identify thin-cap fibroatheromas, and association between laboratory variables and plaque properties.

**Specifications table**

**Table:** 

Subject area	*Medicine*
More specific subject area	*Cardiology-imaging*
Type of data	*figure, Table*
How data was acquired	*Prospective single-center cross-sectional*
Data format	*Raw and analyzed*
Experimental factors	*Coronary angiography, Optical coherence tomography, Flow cytometry, Continuous glucose monitoring*
Experimental features	*Association between arteriosclerosis promoting factor and coronary artery plaque assessed by optical coherence tomography*
Data source location	*Kobe, Japan*
Data accessibility	*Data are within this article*

**Value of the data**

• Patients population enrolled in our research [1].
• Diagnostic ability of CD14^++^CD16^+^ monocytes to identify thin-cap fibroatheromas using receiver operating characteristics curves.
• Association between laboratory variables and plaque properties assessed by optical coherence tomography.

## 1 Data

**Fig. 1:**
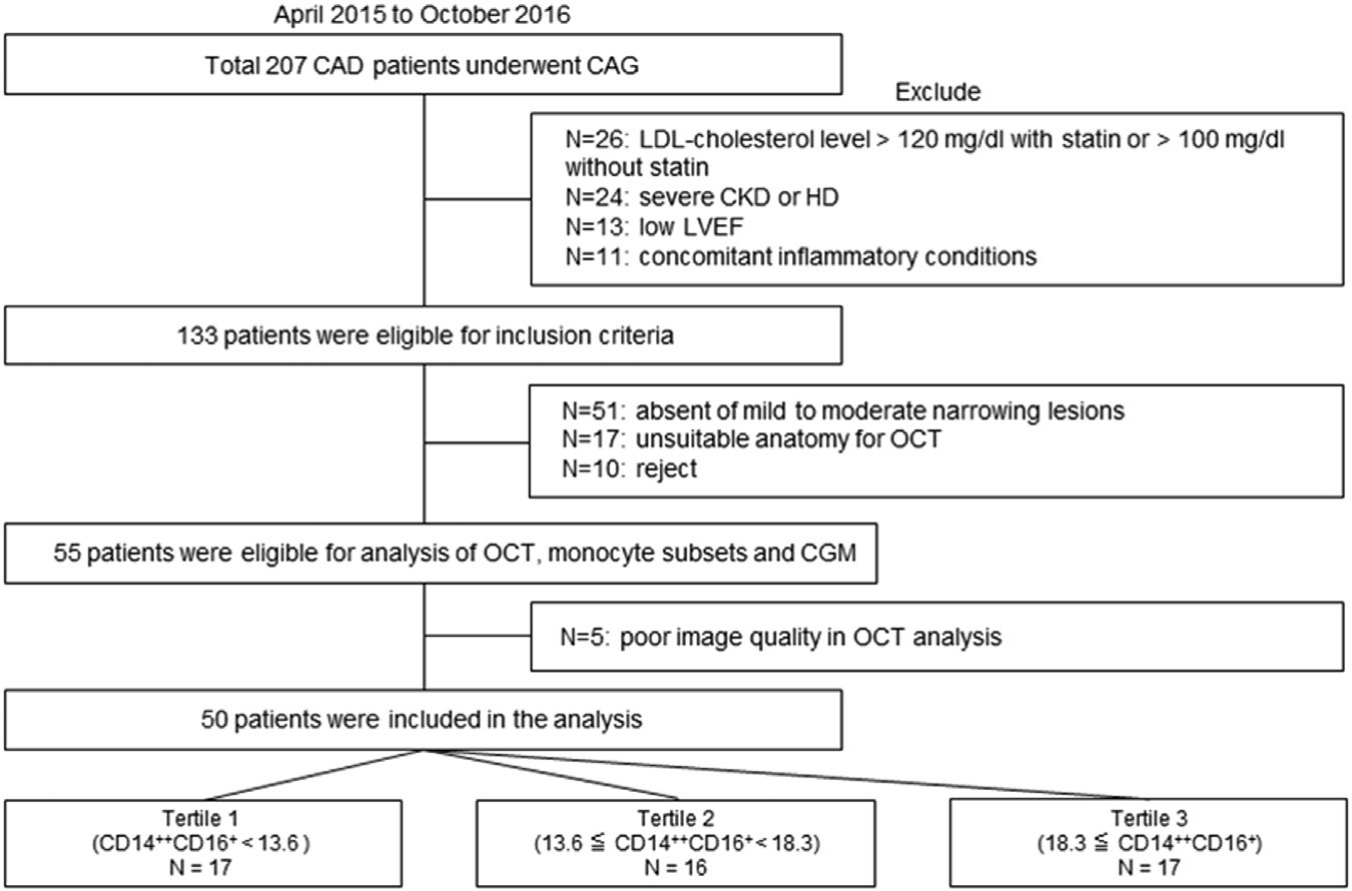
Study population. CKD = chronic kidney disease; LVEF = left ventricular ejection fraction; CGM = continuous glucose monitoring.

**Fig. 2:**
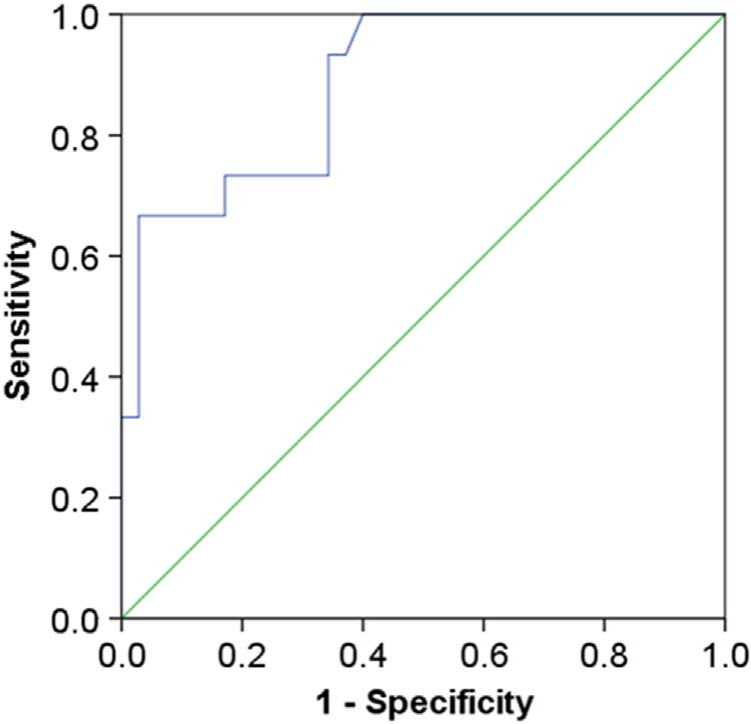
ROC curves for prediction of TCFA. ROC for CD14^++^CD16^+^ monocytes was computed for the prediction of TCFA. ROC = receiver operating characteristic; TCFA = thin-cap fibroatheroma.

All the data shown in this article are supplementary data of our research [1]. Fig. 1 shows flow of study population. Fig. 2 presents the area under the curve (AUC) to predict thin-cap fibroatheroma. Table 1 presents variables measured by the continuous glucose monitoring system. Among total 50 patients, continuous glucose monitoring analysis was performed in 46 patients due to its poor image quality in 4 patients. Table 2 shows association between laboratory variables and plaque properties.

**Table 1:** Variables measured by the continuous glucose monitoring system.

	**Total N = 46**	**Tertile 1** (CD14^++^CD16^+^ monocyte < 13.6) **N = 16**	**Tertile 2** (13.6 ≦ CD14^++^CD16^+^ monocyte < 18.3) **N = 14**	**Tertile 3** (18.3 ≦ CD14^++^CD16^+^ monocyte) **N = 16**	***P*****value**
MAGE, mg/dl	64.6 ± 17.3	56.9 ± 18.6	65.1 ± 17.0	72.0 ± 13.5	0.046
Mean blood glucose, mg/dl	128.9 ± 24.9	125.0 ± 23.4	131.4 ± 28.0	130.5 ± 24.7	0.74
Max blood glucose, mg/dl	220.2 ± 54.2	201.3 ± 60.4	225.6 ± 57.9	234.3 ± 40.4	0.22
Min blood glucose, mg/dl	77.4 ± 25.0	82.8 ± 27.8	73.0 ± 25.6	75.9 ± 21.9	0.60
Time in hyperglycemia, %	32.8 ± 29.7	27.9 ± 33.3	33.7 ± 30.1	37.0 ± 26.6	0.70
Time in hypoglycemia, %	3.65 ± 12.8	1.4 ± 3.8	2.4 ± 4.8	7.0 ± 20.9	0.43

Values are mean ± SD. MAGE = mean amplitude of glycemic excursion.

**Table 2:** Pearson correlation coefficients.

	CD14^++^CD16^+^ monocytes	CRP	LDL cholesterol	HDL cholesterol	HbA1c	MAGE
Lesion length	0.04	0.002	0.12	−0.10	0.07	0.16
Lipid length	0.18	-0.07	0.15	−0.13	0.16	0.28*
Max lipid arch	0.34*	-0.17	0.81	−0.08	0.15	0.34*
Mean lipid arch	0.34*	-0.13	-0.16	−0.04	0.15	0.36*
Lipid index	0.24	-0.10	0.07	−0.11	0.19	0.35*
Calcification length	−0.004	0.05	0.19	0.09	0.03	−0.02
Mean calcification arch	−0.18	-0.02	0.20	−0.005	−0.28	−0.29
Calcification index	−0.13	-0.008	0.19	0.08	−0.07	−0.11
Fibrous cap thickness	−0.51*	0.10	0.09	0.13	−0.19	−0.25

Values are r values. Association between laboratory variables and plaque properties. *P < 0.05. CRP = C-reactive protein; HbA1c = glycated hemoglobin; HDL = high-density lipoprotein; LDL = low-density lipoprotein; MAGE = mean amplitude of glycemic excursion.

## 2 Experimental design, materials and methods

Our research article entitled “Impact of CD14^++^CD16^+^ monocytes on coronary plaque vulnerability assessed by optical coherence tomography in coronary artery disease patients” was a cross-sectional research from single-center prospective registry. Patients admitted with stable coronary artery disease who had undergone coronary angiography were enrolled at Kobe university hospital (Fig. 1). Patients were excluded if they had renal disease (serum creatinine >2.0 mg/dl), low left ventricular ejection fraction (<45%), active infection, inflammatory arthritis, connective tissue disease and malignancies.

Data of coronary angiography, optical coherence tomography, flow cytometry, continuous glucose monitoring was obtained according to the method section of our research [1]. For statistical correlation between two parameters, simple linear correlations were calculated using the method of least squares and by determining the Pearson's correlation coefficient. The AUC was calculated to predict TCFA, with AUC = 0.50 representing no accuracy and AUC = 1.00 indicating maximum accuracy. Analyses were performed using SPSS version 24 (IBM Corp., Armonk, New York). Values of *P* < 0.05 were considered statistically significant.
